# Bites and Some Methods of Taking Them

**Published:** 1898-09

**Authors:** J. Mahoney


ARTICLE VI.

### BITES AND SOME METHODS OF TAKING THEM

J. MAHONEY.

Read before the Students' Society, Victoria Dental Hospital, Manchester, England.

Mr President and Gentlemen: The subject which I have chosen for this evening is one which is very much neglected, although its importance cannot be too highly estimated; so I trust that these few details which I will try to place before you as clearly as possible, will be of use to the student. Not that it will help him much towards obtaining his degree, but one which will be of some practical benefit afterwards.

It is very important to the young practitioner that his first few cases should be successful, and there is nothing which will injure his reputation so much as a badly adjusted artificial plate. Patients may not be able to tell if through some unfortunate condition one is unable to bring to a satisfactory conclusion some operation in filling, etc., but they are able to recognize immediately whether an artificial denture is comfortable or not; and one badly fitting case may mean the loss of many patients, especially at the commencement of a man's career.

I will endeavor in this short paper to describe the difficulties we are likely to meet with in different cases, and the best methods which my short experience has taught me to overcome them.

Although there is apparently a sameness about the taking of the bite in each case we meet, yet there are many little details which must not be overlooked; otherwise we will meet with failures, and it is to these important details I will call your attention.

For our first case will take an edentulous upper and lower, to which we purpose to make a full set of teeth. This is, I think, one of the most difficult in which to obtain a correct bite. A person generally is fairly well on in life by the time he or she has lost all the teeth, and then, as you know, there is much greater movement in the lower jaw than formerly, and this in itself makes it a difficult matter to take the bite.

It is a very good plan to get in the first instance an approximate bite at the visit that you take the impression. This can be done by softening beeswax in warm water and then shape it up roughly to the mouth and get the patient to bite into it. To prevent biting too close, insert an instrument into the center of the wax, although the better plan is to get the patient to close until the lips meet naturally.

After having obtained the models and stearined them, fit into the wax-bite, and fix in position in a slab articulator of either plaster or composition.

Presuming that we are going to make vulcanite plates for this particular case, the next step is to make some hard base on which to set the teeth, and what we must aim at is to use some material which will not alter in shape either with the heat of the mouth or pressure required to obtain the bite, and the best of all materials for this purpose is a plate of vulcanite, vulcanized to duplicates taken from the original models.

This is very quickly done by trimming the duplicates very shallow and drying them, and while hot spread a layer of rubber over them which adheres to the hot model. This method of packing saves a lot of time, as there is no boiling out to be done, for they can be inserted completely in plaster and put into the vulcanizer immediately. By this means you obtain a plate which fits the model perfectly, and which will hold up in position in the mouth much better than any other materials; this is a decided advantage, for if the upper plate will not hold up in position, a wrong bite might be the consequence.

There are other substances which may be used for this purpose, and a very useful one is what is known as "Base plate," sold by S. S. W. These are made up in boxes of one dozen and can be obtained at the depots.

This is extremely useful when there is no time to take duplicates and wait for vulcanizing. They are heated as you would a wax plate and pressed to fit the model. The model ought to be stearined, otherwise it will get rubbed very badly. This base plate will stand the heat and pressure in the mouth quite well, but you cannot get a perfect fitting plate as with vulcanite.

It is customary with some men to set up on the base blocks of composition or wax, but I think it much better to completely set up either the upper or lower, it does not matter which. I have been in the habit of fixing all the teeth on the lower, and the six fronts on the upper, with wax behind to oppose the lower teeth. By this means one is better enabled to notice whether the natural contour of the lips and cheek have been restored, than is possible with simply blocks of wax. The lower teeth also make a perfect imprint in the wax, so that there can be no mistake in fitting together afterwards for the bite in plaster. The great advantage of the rough bite which was taken in wax is now very apparent, because if you obtained it somewhere near you have the teeth probably very nearly correctly placed, and the proper position of the teeth is a good guide in obtaining the right distance between the jaws. Without that temporary bite very probably the position of the teeth will be entirely wrong. They may be either too long or short, or too far in or too prominent, and this will mean a re-setting of both cases, which is a waste of time and trouble.

As before mentioned, the amount of play that exists in the lower jaw of a patient who is edentulous is generally very extensive, and it is best to ascertain the amount of movement that does exist, both laterally and before backwards.

As most of you know, when you ask the patient to bite inside, they generally manage to stick the lower jaw as far out as possible and vice versa. To get the patient to bite in as far as possible, a good plan is to place a flat instrument, such as a spatula, slantways between the teeth; this will cause the jaw to be drawn backwards in closing. As a rule a patient cannot bite very much further back than the natural, so that having obtained the extreme backward position, you can easily get the jaws to close just a little further forward, which will be about the correct position.

If you have the upper teeth placed in the center, and the lower corresponding to them you cannot have the bite very much out laterally.

Something must be said about the size and arrangement of the teeth. In choosing the front teeth, pick out the upper first according to the physique of the patient. Then find a lower set, the canines of which when placed edge to edge with uppers meet between the canines and laterals. This will cause the first upper bicuspid to meet between the first and second lower, and the second upper between the second lower and first molar, which is the arrangement in the natural teeth. The upper bicuspids and molars should also be arranged to curve upwards, the lowest point being about second bicuspid, and the highest the last molar, and the lowers should have the corresponding curve or arch. In other words, the surface of the upper molars and bicuspids should be convex and the lowers concave.

The extent of the curvature of the arch is governed by the depth of the overbite and the length of the cusps on the bicuspids and molars.

The greater the depth of the overbite the more pronounced is the curve of the arch, and vice versa.

It is sometimes rather difficult to obtain this proper curve of the arch, as the tuberosities are often not absorbed, but whenever it is possible that object should be aimed at and straight lines avoided. If you examine a perfect set of natural teeth in situ you will find that if you throw the lower jaw forward until the front teeth meet edge to edge, the lower third molar and the upper twelve year will also be in contact, so that there is pressure at the back as well as at the front. Now if the teeth were arranged in straight lines, this could not be, because there certainly would be a space at the back and all the pressure would be on the front. In an edentulous case, this is a very important point to bear in mind, for if that arrangement is maintained, it will prevent the tilting downward of the upper plate.

I have no doubt some of you have experienced this sort of thing, e. g., patients complaining that when they wished to bite with the front teeth, the upper plate dropped down, which fault I think could have been avoided had some attention been paid to the above mentioned detail.

To make sure that the teeth are in the correct position in order to obtain the above result, a suitable articulation is necessary, and this should represent the movements of the jaw as correctly as possible, so that when the cases are set up, the lower model may be made to move about as the lower jaw does.

The only articulator as far as I am aware, which makes an attempt to represent this movement, is the Bonwill. This gives the necessary movements, but speaking generally, it is not rigid enough, which makes it rather difficult to use.

A very useful and handy articulator is the Ladmore, but this has no side or forward movement. It is a very handy one for working with, as the models can be easily removed and replaced. The distance between them is retained by a small screw which forms a rest. There is a slight movement of the models, however, which is liable to make the bite a little wrong, but this can be counteracted by measuring the distance with a compass and marking that measurement on the back of one model for reference.

Also, if you wish to open the bite a little, this can be done with a few turns of the adjusting screw undeaneath.

It seems to me that if we could have in this articulator the lateral and forward movements, we should have a well nigh perfect articulator.

What has been said about an edentulous case will apply in a more or less modified condition, to most other cases, so that there will be no need for repetition.

The next case we shall consider will be an upper with one molar standing, to which we intend to make a vulcanite or metal plate; and let us suppose that there are not sufficient teeth lost in the lower to need a plate-making.

To this molar we should naturally fit a band, and I will try to show you how the presence of this molar and band may be the cause of an incorrect bite. This case should be set up with the six fronts and wax at the back to oppose the lower teeth. When the plate is being tried in, it is quite possible that on account of the band fitting badly, or fitting too tightly, the plate tilts down on the opposite side, and it is to this titling that I would call your attention, for if the plate does not fit up into position accurately the bite is sure to be short on that side which tilts; to remedy which would mean removing and lowering the teeth on that side. So, before we get the patient to bite into the wax, we must make sure that the plate goes well up into position and remains there while the bite is being taken. If it is found impossible to keep the plate in position, a good plan is to put an additional quantity of wax on the side opposite to the molar, keeping it rather harder than the other side, and this will force it up into position; or an instrument might be pressed up on that side into the wax.

As another example, we might take this same case which we have just considered, with this difference, that there are sufficient teeth out in the lower to need a case some time, but which for various reasons we do not wish to make at the time the upper plate is made; say all the back teeth on one side are gone.

Take a good impression of the lower, all the same, just as if you intended making a case to it, and set up in this either in composition or wax, the back teeth, so that there will be something solid to oppose the wax in the upper. This will enable you much more easily to fix the models accurately together afterwards. Also, if a good impression were taken of the lower, there would be less likelihood of making the upper so that there is scarcely sufficient room for the lower teeth, through bringing the uppers too low at the back. This, of course, would not be noticed until a lower plate came to be made.

I think we have considered sufficient examples in detail for our purpose, and now I should like to call your attention to a few points which may occur in nearly every case.

The titling of upper plates, as before mentioned, is often the cause of a lot of trouble, even in cases where there are several teeth standing, unless there are teeth on both sides to which we can fasten bands. It will be hardly necessary to enumerate the cases in which this fault is likely to occur. The best thing to do is to be prepared for the trouble, and, if possible, avoid or counteract it.

Always take good impressions whether a case is to be made or not.

A frequent cause of wrong bite is because very little care is bestowed on the impression of opposing teeth when no denture is required for the portion. The result is that the teeth come out sucked and, therefore, shorter, which necessitates sometimes a great amount of grinding when the finished plate is inserted, and often molars and bicuspids are put on a case when there is only room for flat teeth. In taking impressions it is often necessary to make a special tray, and when that is the case one is likely to bestow very little care on the first one, so that one ought always to try the cases in with the plates fitted to the good impression; because if fitted to the first and probably rough one, they will not go down into position on the second, consequently a wrong bite will be the result. This is, I think, another very frequent cause of trouble.

When trying in plates always have the models handy, and after the patient has biten into the wax put the cases on it, and see if they meet together as they do in the mouth, and if there is any discrepancy allow for it and make a note or mark on the model as a reminder. It may be that the teeth on the model seem shorter or longer; if the former, you may depend that it is because the impression was removed before being set, and if longer, very probably the case or cases were not properly in position. Always have a good imprint in the wax of opposing teeth so that there is no doubt whatever, which is the correct position in which to fix them for the articulator.

In trying in cases there are many points which should be noticed, such as whether the bands fit the natural teeth as they do on the model, and also their suitability in shape to the teeth which they clasp; but this hardly comes within the scope of this paper.

One gentleman remarked to me once that if he were in a busy practice he would never try his cases in. I should think the busier he became the more need there would be to do so, as he would be less able to notice any peculiarity which might exist, in the hurry of taking an impression. How he would take a correct bite at the same time, I cannot make out.

It is quite evident that too much care cannot be bestowed on this, the most lucrative branch of the dental profession, and the amount of extra time spent in obtaining the bite will, in the end, be repaid threefold,—*Indiana Dental Journal.*

